# Thermoneutral Housing Enables Studies of Vertical Transmission of Obesogenic Diet-Driven Metabolic Diseases

**DOI:** 10.3390/nu15234958

**Published:** 2023-11-29

**Authors:** Jennifer L. Wayland, Jessica R. Doll, Matthew J. Lawson, Traci E. Stankiewicz, Jarren R. Oates, Keisuke Sawada, Michelle S. M. A. Damen, Pablo C. Alarcon, David B. Haslam, Andrew T. Trout, Emily A. DeFranco, Corie M. Klepper, Jessica G. Woo, Maria E. Moreno-Fernandez, Marialena Mouzaki, Senad Divanovic

**Affiliations:** 1 Immunology Graduate Program, University of Cincinnati College of Medicine, Cincinnati, OH 45220, USA; 2 Medical Scientist Training Program, University of Cincinnati College of Medicine, Cincinnati, OH 45220, USA; 3 Division of Immunobiology, Cincinnati Children’s Hospital Medical Center, Cincinnati, OH 45229, USA; 4 Division of Infectious Diseases, Cincinnati Children’s Hospital Medical Center, Cincinnati, OH 45229, USA; 5 Department of Pediatrics, University of Cincinnati College of Medicine, Cincinnati, OH 45220, USA; 6 Department of Radiology, Cincinnati Children’s Hospital Medical Center, Cincinnati, OH 45229, USA; 7 Department of Radiology, University of Cincinnati College of Medicine, Cincinnati, OH 45220, USA; 8 Department of Obstetrics and Gynecology, University of Cincinnati College of Medicine, Cincinnati, OH 45220, USA; 9 Division of Gastroenterology, Hepatology and Nutrition, Cincinnati Children’s Hospital Medical Center, Cincinnati, OH 45229, USA; 10 Division of Biostatistics and Epidemiology, Cincinnati Children’s Hospital Medical Center, Cincinnati, OH 45229, USA; 11 Center for Inflammation and Tolerance, Cincinnati Children’s Hospital Medical Center, Cincinnati, OH 45229, USA

**Keywords:** developmental origin of health and disease (DOHaD), inflammation, intrauterine programming, amniotic fluid, type 2 diabetes, metabolic dysfunction-associated steatotic liver disease (MASLD), pregnancy, stillbirth, high-fat diet

## Abstract

Vertical transmission of obesity is a critical contributor to the unabated obesity pandemic and the associated surge in metabolic diseases. Existing experimental models insufficiently recapitulate “human-like” obesity phenotypes, limiting the discovery of how severe obesity in pregnancy instructs vertical transmission of obesity. Here, via utility of thermoneutral housing and obesogenic diet feeding coupled to syngeneic mating of WT obese female and lean male mice on a C57BL/6 background, we present a tractable, more “human-like” approach to specifically investigate how maternal obesity contributes to offspring health. Using this model, we found that maternal obesity decreased neonatal survival, increased offspring adiposity, and accelerated offspring predisposition to obesity and metabolic disease. We also show that severe maternal obesity was sufficient to skew offspring microbiome and create a proinflammatory gestational environment that correlated with inflammatory changes in the offspring in utero and adulthood. Analysis of a human birth cohort study of mothers with and without obesity and their infants was consistent with mouse study findings of maternal inflammation and offspring weight gain propensity. Together, our results show that dietary induction of obesity in female mice coupled to thermoneutral housing can be used for future mechanistic interrogations of obesity and metabolic disease in pregnancy and vertical transmission of pathogenic traits.

## 1. Introduction

Obesity is an unabated pandemic [1]. Obesity-associated low-grade chronic inflammation contributes to insulin resistance and glucose dysmetabolism, leading to higher risk for development of type 2 diabetes (T2D) [2]. In parallel, obesity promotes lipid accumulation in hepatocytes, resulting in liver tissue inflammation as well as development and progression of metabolic dysfunction-associated steatotic liver disease (MASLD) [3]. Given the rapid increase in prevalence of obesity and the surge in obesity-associated metabolic diseases [1], an improved understanding of factors that predispose to development of obesity is needed.

Maternal obesity is associated with detrimental health outcomes for both the mother and offspring. In the mother, obesity in pregnancy increases the risk of complications, including pregnancy loss, gestational diabetes mellitus, maternal hypertension, preeclampsia, and C-section delivery [4]. In the offspring, both clinical and experimental studies indicate that offspring of parents with obesity are more likely to have obesity themselves [5] and also have higher risk for developing T2D [6] and MASLD [7,8,9,10] independent of diet [11]. Given the recent rapid increase in incidence of obesity, genetic mutations alone can only partially explain its heritability [12]. Hence, the discovery of factors that shape non-genetic “vertical transmission” of obesity is critical.

Mice are the most commonly used experimental animal in biomedical research [13], given their genetic and physiologic similarities to humans [14]. However, difficulties in inducing diet-induced obesity in wild-type (WT) female C57BL/6 mice (the most common inbred strain of laboratory mice used [15]) have greatly hindered research efforts to interrogate the vertical transmission of obesity. Specifically, the induction of severe diet-induced obesity (DIO) and metabolic disease (e.g., glucose dysmetabolism, MASLD) is achievable in C57BL/6 WT male mice through obesogenic diet feeding, including high-fat diet (HFD) [16,17,18]. Conversely, HFD feeding alone in C57BL/6 WT female mice has only mild effects on obesity and metabolic health [19,20,21,22,23,24,25,26]. Other diets (e.g., high-fat/high-sugar, cafeteria/”junk food” diets) used to better mimic human dietary intake induce more weight gain in female mice than HFD but still fail to drive metabolic dysfunction across multiple organ systems [27,28,29,30,31,32,33,34]. Thus, it is not surprising that sex represents a major biological variable in experimental mouse models of clinically relevant obesity. To circumvent these issues, the field has turned to surgical (ovariectomy) [35] or genetic (deletion of satiety regulator leptin or its receptor [*ob/ob, db/db*] [36,37,38]) modifications to induce severe DIO in female mice. However, ovariectomy impacts fertility [36,37,39,40], while *ob/ob* and *db/db* mice exhibit substantial developmental abnormalities [40,41], and *ob/ob* mice exhibit suppressed innate and adaptive immune responses [42]. Combined, such limitations have resulted in the lack of a consensus on a more “human-like” model of severe obesity and metabolic disease in female mice and have greatly restricted efforts to study how maternal obesity impacts pregnancy and offspring health. 

Mechanistic studies have been undertaken to study various postulated components of vertical transmission of obesity and metabolic disease, including epigenetic marks in sperm and oocytes [43,44], oxidative and lipotoxic stress on the placenta [45,46], shared environmental factors such as food availability or nutrition [47], and gut microbiome [8,48,49,50,51,52,53]. Another notable postulated component is intrauterine programming—in which the gestational environment imparts permanent changes in the offspring [54] via increased levels of systemic inflammatory cytokines and chemokines in mothers with obesity [55,56,57], nutritional stimuli [58], or direct effects of altered immune cell inflammatory response at the maternal–fetal interface [59,60,61,62]. However, the lack of a consistent mouse model to study maternal obesity has hampered elucidation of contributions of specific mechanisms to offspring health.

Animal facilities are predominantly maintained at “room temperature” (19–22 °C) for the comfort of the human work environment [63,64]. The thermoneutral (TN) zone, or temperature of metabolic homeostasis, for C57BL/6 mice is between 30–32 °C [20]. Thus, traditional housing conditions at “room temperature” expose mice to chronic thermostress (TS) conditions. Specifically, in comparison to TS, TN housing is associated with significant decreases in physiologic (e.g., basal heart rate and mean arterial blood pressure [65,66]) and metabolic (e.g., food intake and basal metabolic rate [67]) parameters and increases in inflammatory (e.g., serum cytokine levels [20]) parameters at the basal state. Notably, TN housing increases adipocyte size in murine inguinal white adipose tissue (iWAT) [68] and causes mouse brown adipose tissue (BAT) to closely resemble human BAT [69]. Such modulation of mouse physiology has allowed TN housing to improve mimicry of many human disease models, including cancer [64,70,71,72,73], atherosclerosis [21,74], asthma [75], food allergy [76], and liver disease [77]. Specifically, TN housing allows for induction of HFD-induced severe obesity and amplifies severity of obesity-associated sequelae (e.g., glucose dysmetabolism, hepatocellular damage, influenza virus infection pathology) in WT female C57BL/6 mice [20,78]. However, whether the coupling of TN housing with HFD enables studies of vertical transmission of obesity is not known.

Here, via syngeneic mating of WT obese female and lean male C57BL/6 mice under physiological conditions (i.e., thermoneutral housing temperature) that more closely mimic obesity in humans, we studied whether our new model represents a tractable approach to investigate maternal-specific obesity contributions to offspring health. We found that maternal obesity decreased neonatal survival and increased adiposity. Further, offspring of obese dams exhibited increased obesity and metabolic disease severity compared to those of lean dams after HFD feeding. Manipulation of offspring microbiome did not alter the effects of maternal diet on offspring susceptibility to weight gain and dysmetabolism following HFD feeding. When investigating obesity-associated inflammation, maternal obesity was sufficient to amplify proinflammatory skewing of both systemic and gestational environments in the mother and augmented offspring in utero inflammatory responsiveness. Further, to correlate our experimental model with humans, data were collected and analyzed from a cohort of mothers with and without obesity and their respective infants. In mothers with obesity compared to mothers without obesity, we found increased maternal serum proinflammatory cytokines and increased child’s propensity to early-life weight gain, mirroring findings from mouse studies. Together, our data demonstrate that TN housing, coupled with HFD feeding-induced obesity in female mice, represents a novel and improved experimental model that can be used to interrogate vertical transmission of obesity traits observed in humans.

## 2. Materials and Methods

### 2.1. Mice

All mice used were on a WT C57BL/6J background (Jackson Laboratories, Bar Harbor, ME, USA). Mice were housed at Cincinnati Children’s Hospital Medical Center (CCHMC) in a specific pathogen-free facility maintained at 22 °C, with free access to autoclaved chow diet (fat 13.5% kcal, carbohydrate 59% kcal, protein 27.5% kcal; LabDiet 5010, St. Louis, MO, USA) and water. At 6 weeks of age, mice were moved to a separate specific pathogen-free room maintained at T_N_ (30 °C). Food and water were replaced weekly. All animal care was provided in accordance with the Guide for the Care and Use of Laboratory Animals. All studies were approved by the Cincinnati Children’s Hospital Medical Center Institutional Animal Care and Use Committee (IACUC).

### 2.2. Maternal Obesity Model and Mouse Study Design

To investigate pregnancy outcomes, all breeding mice were housed at 30 °C (T_N_). After 2–4 weeks of acclimation, females were fed either an irradiated high-fat diet (HFD; fat 60% kcal; Research Diets #D12492, New Brunswick, USA) or chow diet (CD). Male mice were housed in separate cages and were fed CD. Females were kept on their respective diets for at least 6 weeks prior to breeding. To avoid male HFD consumption, male mice were introduced once weekly into female cages overnight for mating. Female mice were checked for the presence of a sperm plug and weighed the following day. 

#### 2.2.1. Experimental Design: Pregnancy and Neonatal Offspring Health

**Figure 1:**
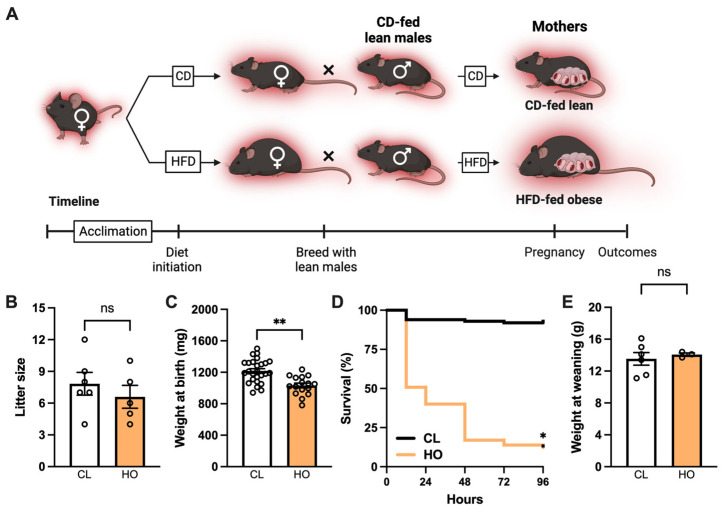
Maternal obesity at thermoneutrality leads to adverse neonatal outcomes. (**A**) Approach used to study maternal obesity in female C57BL/6 mice housed at thermoneutral temperature (red background) and fed chow (CD) or high-fat diet (HFD). Maternal groups are CD-fed lean (CL) and HFD-fed obese (HO). (**B**) Number of pups per litter (CL *n* = 6, HO *n* = 5). (**C**) Pup weight at birth (CL *n* = 26, HO *n* = 16). (**D**) Pup survival from 24–96 h after birth (CL *n* = 29, HO *n* = 39). (**E**) Male pup weight at weaning (CL *n* = 6, HO *n* = 3). (**B**,**C**,**E**) Unpaired *t*-test. ** *p* < 0.01; ns = not significant. (**D**) Log-rank test, curves are significantly different, * *p* < 0.0001.

Dams were classified as CD-fed lean (average weight 24.8 g) or HFD-fed severely obese (>12 weeks of HFD feeding, weight > 35 g) based on their pre-pregnancy weight (Figure S1A and Figure 1A). Pregnant females approaching term pregnancy were monitored daily. When dystocia was noted, the animal was immediately euthanized. Upon presence of pups in the cage, the number of total and living pups and weight of each living or deceased pup were recorded. Litters were monitored for the next three days for survival. Dams continued their assigned diet throughout pregnancy and nursing. Offspring were weaned to chow diet at 3–4 weeks of age. Randomly selected litters were weighed at weaning.

#### 2.2.2. Experimental Design: Offspring Health at Baseline

**Figure 2:**
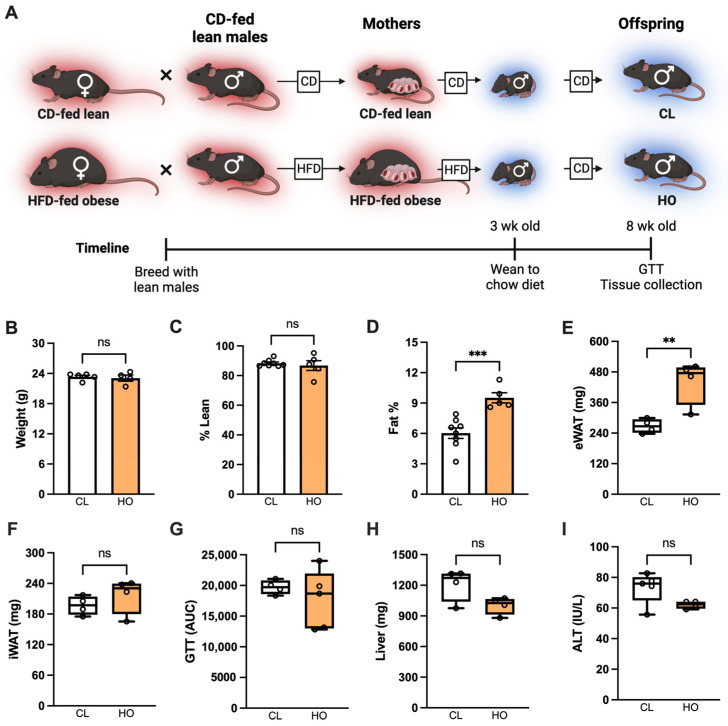
Offspring of HFD-fed mothers weaned to CD have changes in adiposity but no metabolic disease. (**A**) Schematic of approach. Red background denotes thermoneutral housing temperature, and blue background denotes thermo-stressed housing. (**B**) Weight of CD-fed offspring at 8 weeks of age. Groups are male offspring of dams that were CD-fed lean (CL, *n* = 5) or HFD-fed obese (HO, *n* = 4). (**C**) Lean mass percentage of offspring (CL *n* = 8, HO *n* = 5). (**D**) Fat percentage of offspring (CL *n* = 8, HO *n* = 5). (**E**) eWAT mass of offspring (CL *n* = 4, HO *n* = 4). (**F**) iWAT mass of offspring (CL *n* = 4, HO *n* = 4). (**G**) Area under curve (AUC) from two-hour glucose tolerance test (GTT) (CL *n* = 4, HO *n* = 5). (**H**) Liver weight of offspring (CL *n* = 4, HO *n* = 4). (**I**) ALT of offspring (CL *n* = 4, HO *n* = 4). (**B**–**I**) Unpaired *t*-test. ** *p* < 0.01, *** *p* < 0.001, ns = not significant.

To investigate the effect of maternal HFD and obesity on offspring fed CD, male offspring of CD-fed lean and HFD-fed obese mothers were weaned to CD and were used for studies at 8 weeks of age (Figure 2A). 

#### 2.2.3. Experimental Design: Offspring Health during HFD Challenge

**Figure 3:**
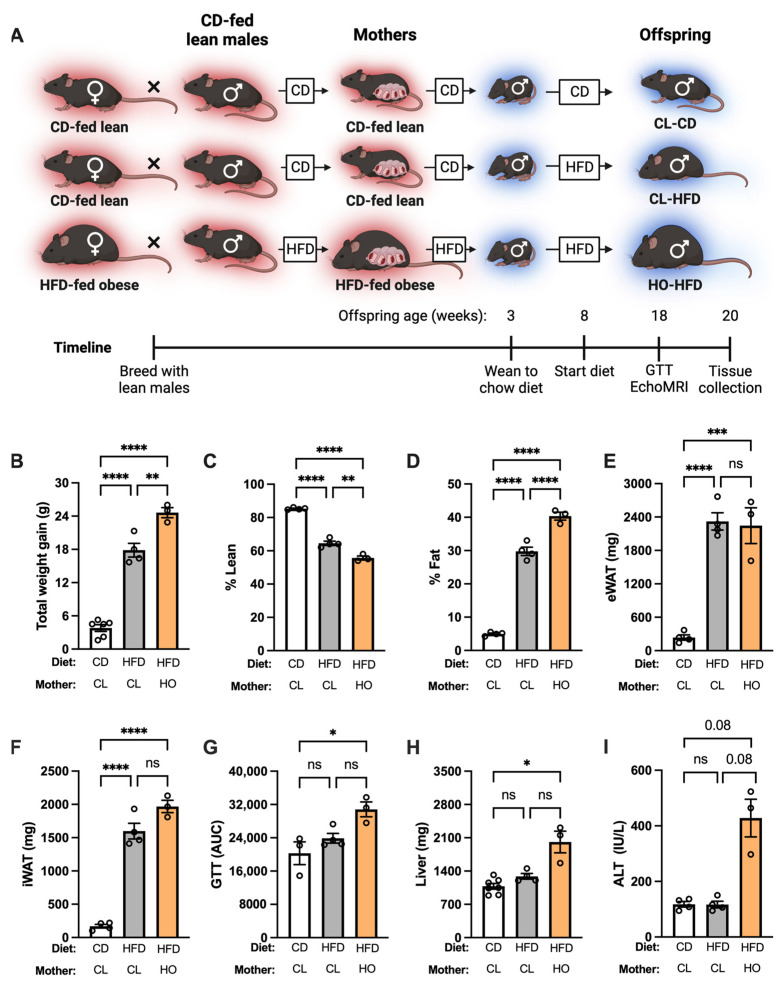
HFD challenge causes more severe metabolic disease in offspring of HFD-fed obese mothers. (**A**) Schematic of approach. Red background denotes thermoneutral housing temperature, and blue background denotes thermo-stressed housing. Unless otherwise noted, CD-CL, *n* = 4; HFD-CL, n = *4*; HFD-HO, *n* = 3. (**B**) Weight gain of male offspring after 12 weeks on diet. Groups are offspring of dams that were CD-fed lean (CL) or HFD-fed obese (HO). CD-CL, *n* = 8. (**C**) Lean mass percentage (lean mass divided by total body weight) of offspring. (**D**) Fat percentage of offspring. (**E**) eWAT mass of offspring. (**F**) iWAT mass of offspring. (**G**) Area under curve (AUC) from two-hour glucose tolerance test (GTT). CD-CL, *n* = 3. (**H**) Liver weight of offspring. CD-CL, *n* = 7. (**I**) ALT of offspring. (**B**–**I**) One-way ANOVA with Dunnett’s multiple comparisons test. * *p* < 0.05; ** *p* < 0.01, *** *p* < 0.001, **** *p* < 0.0001, ns = not significant.

To investigate the effect of maternal HFD and obesity on offspring fed HFD, male offspring of CD-fed lean and HFD-fed obese mothers were weaned to CD. At 8 weeks of age, male offspring were either switched to HFD feeding for 12 weeks or maintained on CD as a control (Figure 3A). Terminal harvest occurred at 12 weeks after diet change.

#### 2.2.4. Experimental Design: Microbiome Manipulation

To determine the effect of microbiome manipulation, offspring of CD-fed or HFD-fed dams were used. All offspring were weaned to CD at 3–4 weeks of age, and fecal samples were obtained at 8 weeks of age (Groups (a) and (b), Figure S2A). At 9 weeks of age, offspring of HFD-fed mothers were either given water supplemented with antibiotics (0.5 mg/mL Vancomycin, 0.5 mg/mL Neomycin, 0.125 mg/mL Polymyxin B) or regular drinking water as control for 3 weeks, then allowed to recover with no treatment for 2 weeks. After the recovery period, a second set of fecal samples was obtained from antibiotic-treated and untreated mice (Groups (c) and (d), Figure S2A). All mice were then fed HFD for 12 weeks. A final set of fecal samples was obtained after 6 weeks of HFD feeding (Groups (e) and (f), Figure S2A). Terminal harvest occurred at 12 weeks after diet change.

### 2.3. In Vivo and Serum Assays (GTT, EchoMRI, Hemavet, ALT)

EchoMRI and GTT were performed after 10 weeks of dietary manipulation for all offspring studies except those described in Section 2.2.2, in which they were performed when offspring were 8 weeks old. Following overnight fast, glucose tolerance levels were determined by injecting mice with 10 μL of a 10% dextrose solution per gram of body weight, and glucose levels were quantified kinetically at 0, 20, 40, 60, 90, and 120 min. Total body fat, lean, and water mass were determined by nuclear magnetic resonance (Whole Body Composition Analyzer; EchoMRI LLC, Houston, TX, USA). Percent fat mass and percent lean mass measured by EchoMRI were calculated by dividing fat mass or lean mass, respectively, by total body weight. Complete blood count was performed immediately after terminal tissue collection using a Hemavet automated hematology unit (Drew Scientific, Miami Lakes, FL, USA). Serum alanine transaminase (ALT) was determined according to manufacturer protocol (Catachem Inc., Oxford, MS, USA). Sample absorbance was read once per minute for five minutes to quantify enzyme activity. The calibrator mixtures Catatrol™ I and Catatrol™ II (Catachem Inc.) were used as positive controls for the ALT assay.

### 2.4. Cytokine and Endotoxin Quantification

For quantification of cytokine production in mice, in vivo cytokine capture assays were employed as previously described [20,79,80]. Briefly, cytokines were detected using In Vitro Cytokine Capture Assay (IVCCA) employing biotinylated capture antibodies (IL-6 (MP5-32C11), TNFα (TN3-19)), detection antibodies, and recombinant protein mouse standards (eBioscience Inc., San Diego, CA, USA). Biotinylated capture antibodies were injected intraperitoneally, followed by LPS injection 3 h later and terminal serum collection after an additional 4 h. 

For quantification of cytokine production in vitro, levels of IL-6 in supernatant were determined via ELISA according to manufacturer protocol (BD OptELIA, Franklin Lakes, NJ, USA). 

For quantification of cytokine levels in human serum, U-PLEX assay (Meso Scale Discovery) was used according to manufacturer protocol. The assay was read using a Meso Sector S 600 instrument (Meso Scale Discovery, Rockville, MD, USA). 

For endotoxin quantification, LAL assay (Lonza, Basel, Switzerland) was performed on serum or AF according to manufacturer protocol.

### 2.5. Mouse Embryonic Fibroblast (MEF) Isolation and Culture

MEFs were collected from CD-fed lean and HFD-fed obese dams on day 14 of pregnancy. Embryos were isolated, and heads and livers were discarded. The remaining tissue was mechanically minced and digested in 2× trypsin to create a single-cell suspension, then plated. Upon reaching confluence, MEFs were passaged and plated at 200,000 cells per well. Cells were rested for 24 h, washed to remove non-adherent cells, and then stimulated with ultrapure LPS (Invivogen, San Diego, CA, USA; 100 ng/mL) or culture media for 24 h, followed by supernatant collection and subsequent cytokine quantification.

### 2.6. Flow Cytometry

Flow cytometry was performed on tissue from offspring of CD-fed lean and HFD-fed obese dams after a 12-week HFD challenge. Single-cell suspensions were derived from epididymal white adipose tissue (eWAT) homogenate and stained with directly conjugated monoclonal antibodies for surface markers. Data collection and analysis were performed as previously described [81]. Briefly, cells were stained with Live/Dead stain (Zombie UV Dye: Biolegend, San Diego, CA, USA) and with directly-conjugated monoclonal antibodies to the following panel: Gr1-APC, CD11c-BV711, F4/80-AF700, TCRβ-APC-ef780, B220-BV605, TNFα-BV650, IL-6-PE, CD45-PEDazzle594, CD11b-ef450, IL-17A-PerCP-Cy5-5, IFNγ-PE-Cy7, CD8-BV510, NK1.1-AF488 (all antibodies from eBioscience). Flow cytometry data were then collected using an LSR Fortessa (BD) flow cytometer and analyzed using FlowJo software (v10.8.2).

### 2.7. Shotgun Metagenome Sequencing and Data Analysis

Bacterial DNA was isolated from fecal material obtained at the time points described in Section 2.2.4. DNA was extracted from one or two fecal pellets using the Power Fecal DNA Isolation Kit^®^ by MO BIO^®^ per kit instructions. DNA concentration was measured using Qbit^®^. Amplified library generation was performed using the Nextera XT^®^ protocol according to the manufacturer’s recommendations, and sequencing was performed to obtain 150 bp DNA paired-end reads to a depth of approximately 20 million base pairs per sample using an Illumina NovaSeq 6000 sequencing machine (Illumina Corp., San Diego, CA, USA). Raw sequence reads were extracted and demultiplexed using the Illumina program bcl2fastq. Raw reads were then filtered and trimmed for quality control using the program Sickle [82]. Trimmed reads were aligned using Kraken [83] to a custom microbial genome database that includes all RefSeq bacterial, fungal, parasitic, and viral genomes supplemented with additional bacterial and fungal genome sequences from the National Center for Bioinformatics to determine quantitative genus and species abundance for more than 40,000 microbial species genomes. An exact sequence read match of k-mer length 32 was used in Kraken to assign reads to the lowest common ancestor. Normalization of count data to the lowest number of total reads mapped among the samples was performed using rrarefy with the Vegan package in R to give the relative abundance at both the genus and species level [84]. Principal component analysis was performed on a Bray–Curtis distance matrix calculated from normalized species abundance data using the ade4, Vegan, and factoextra packages in R and the FactoExtra package. Statistical significance of differences in overall microbiome composition was determined using multiresponse permutations procedures (MRPP), a form of PERMANOVA [85]. Differential species and genus abundance between groups was determined by pairwise Wilcox rank sum test with false-discovery rate (FDR) correction for multiple testing. Fold-change and log2 fold-change were calculated with the gtools package in R.

### 2.8. Proteomics

Given the suggested link between inflammatory environment and vertically transmitted modification in inflammatory responsiveness [54,86], the intrauterine environment in obese and lean mothers was further characterized by protein mass spectrometry. Amniotic fluid (AF) was collected at day 16 of gestation from HFD-fed obese pregnant and CD-fed lean pregnant mice housed at T_N_. Collected AF was immediately frozen in liquid nitrogen and shipped for analysis. Mass spectrometry and subsequent analysis were performed at Pacific Northwest National Laboratory as previously described [87]. Briefly, AF samples were subjected to a low-efficiency albumin depletion with a spin column, and the sample was analyzed by liquid chromatography-tandem mass spectrometry (LC-MS/MS). LC-MS/MS measurements were performed using a label-free approach utilizing MS peak intensities for quantification. Protein identification and quantification was performed using MaxQuant software v2.4.2.0 [88]. 

### 2.9. Human Study Design

Human data were taken from a prospective birth cohort study performed at the University of Cincinnati (UC), The Christ Hospital (TCH), and Cincinnati Children’s Hospital Medical Center (CCHMC). Pregnant women were approached for recruitment during their regular clinic visits with the obstetrician. Enrollment took place between September 2020 and April 2023. Inclusion criteria were singleton pregnancy at or beyond the 28th week of gestation. Only women ages 18 years or older were approached. Exclusion criteria were inability to provide informed consent, inability to undergo Magnetic Resonance Imaging (MRI), active maternal or fetal health issues beyond the metabolic conditions investigated in this study (e.g., obesity, pre-existing MASLD, pre-existing T2DM, gestational diabetes), use of medications that can predispose to hepatic steatosis (e.g., corticosteroids) and use of alcohol or other drugs of abuse at time of enrollment. All study visits took place after informed consent was signed. The study protocol was approved by the Institutional Review Boards of all three institutions (UC, TCH, and CCHMC). 

Clinical data (gestational age at enrollment, anthropometrics at enrollment and pre-pregnancy, diagnoses (type 2 diabetes mellitus, gestational diabetes; GDM, etc.), medication use) and demographic data (e.g., maternal age, race, ethnicity) were collected from the electronic medical record (EMR). Pre-pregnancy obesity was defined as a pre-pregnancy body mass index (BMI) > 30 kg/m^2^. Pregnant women were asked to return fasting for a research visit at or beyond the 32nd week of gestation. The visit included the collection of anthropometric data (weight and height), gestational age at imaging, abdominal and fetal MRI, and blood collection for testing. Subcutaneous fat thickness of the fetal abdominal wall was measured by an expert radiologist using a single linear measurement anterior to the liver using a 3 Tesla research MRI (Ingenia, Philips; Best, The Netherlands). Labs included a fasting lipid profile, serum aminotransferase levels, and cytokine analysis (see Section 2.4). 

Following delivery and at 9 months of age, offspring information, such as birth weight, length, and head circumference, as well as infant sex and race/ethnicity, were collected. To determine whether infant weight early in life was affected by maternal weight, the change in weight-for-length Z-score, a standard WHO parameter denoting standard deviations from median [89], was analyzed. 

### 2.10. Statistical Analysis

For statistical analysis, normality and lognormality tests and parametric tests were employed using Graphpad Prism software version 9. A 2-tailed Student’s *t*-test or 1-way ANOVA with multiple comparisons was used based on the number of experimental groups. Statistical analysis was completed using Prism version 9 (GraphPad Software Inc., Boston, MA, USA). Unless otherwise indicated, values are represented as means ± SEM. A *p*-value less than 0.05 was considered significant.

## 3. Results

### 3.1. Maternal Obesity Impacts Neonatal Offspring Health in Mice

The use of thermoneutral housing modifies common physiological responses (e.g., heart rate, blood pressure, catecholamine release, basal metabolic rate) in mice [63]. In fact, the potential clinical relevance of such changes in modelling disease pathogenesis has been widely studied with strong success (e.g., cancer, asthma, allergy, inflammation, chronic stress) [68,90]. We have previously shown that the combination of HFD with thermoneutral housing (TN) creates a human-like DIO phenotype in male mice (e.g., induction of hepatic fibrosis [77], induction of atherosclerotic plaques [21], and discovery and recognition of rare immune cells that promote progression of MASLD [20]). Of note, we have also further expanded upon these findings and have shown that female mice housed at TN can also achieve robust obesity and obesity-associated metabolic disease severity [20]. Notably, comparison of obesity and associated parameters achieved at TN with that of other traditionally used models demonstrates the ability of TN housing to promote a greater degree of obesity and metabolic disease in WT female mice (Table 1 [20]). 

Thus, we next asked whether our model could be used to study the effect of maternal obesity on maternal and offspring health. To begin to answer this question, we studied maternal outcomes in CD-fed lean (CL) and HFD-fed obese (HO) female mice housed at T_N_ (Figure 1A). 

As expected, pre-pregnancy weight was significantly higher in HO dams than in CL dams (Figure S1A). Gestational weight gain (Figure S1B) and frequency of dystocia events were not different between the groups of dams (Figure S1C; Fisher’s exact test *p* = 0.67). 

Following studies of maternal pregnancy outcomes in obesity, we asked whether neonatal health was affected by severe maternal obesity. The number of pups per litter was not different between CL and HO dams (Figure 1B); however, offspring of HO dams had lower birth weight compared to offspring of CL dams (Figure 1C). In conjunction with lower birth weight, litters from HO dams had significantly increased offspring mortality in the first 4 days of life compared to litters from CL dams (Figure 1D). Additionally, in representative litters weighed at weaning, surviving offspring of HO dams were not different in weight than offspring of CL dams (Figure 1E). 

### 3.2. Absence of Metabolic Dysfunction at Baseline in Offspring of HFD-Fed Obese Dams

We next examined whether maternal obesity could shape offspring health when offspring were fed CD (Figure 2A).

At 8 weeks of age, despite similar body weight among offspring of CL and HO dams (Figure 2B), notable differences in body composition were observed. Offspring of HO dams had similar lean mass and increased total body adiposity compared to offspring of CL dams (Figure 2C,D). The weight of the visceral epididymal white adipose tissue (eWAT) depot, but not the subcutaneous inguinal WAT (iWAT) depot, was significantly increased in offspring of HO dams compared to offspring of CL dams (Figure 2E,F). However, despite altered adiposity, metabolic parameters such as glucose metabolism measured by GTT (Figure 2G), liver weight (Figure 2H), and hepatocellular damage measured by ALT (Figure 2I) were not different. 

### 3.3. Development of Metabolic Dysfunction in Offspring of HFD-Fed Obese Dams after HFD Feeding

We next examined whether maternal obesity could shape offspring health when offspring were fed HFD. Offspring of CL mothers fed HFD for 12 weeks (CL-HFD, Figure 3A) displayed significant weight gain, decreased lean mass, and increased fat mass compared to offspring of CL mothers fed CD (CL-CD; Figure 3A–D). 

This was also reflected in increased weight of eWAT and iWAT fat depots in CL-HFD compared to CL-CD (Figure 3E,F). However, CL-HFD offspring did not develop any alterations in glucose metabolism (Figure 3G), liver weight (Figure 3H), or ALT (Figure 3I) compared to CL-CD controls. In offspring of HO mothers fed HFD for 12 weeks (HO-HFD, Figure 3A), HFD feeding resulted in significantly greater weight gain, increased fat mass gain, and greater adiposity compared to offspring of CL-HFD (Figure 3B–D). No significant difference in eWAT or iWAT weight was observed in HO-HFD compared to CL-HFD (Figure 3E,F). In contrast to CL-HFD, HO-HFD displayed significantly increased glucose dysmetabolism (Figure 3G), liver weight (Figure 3H), and a trend toward increased liver damage (ALT) (Figure 3I, *p* = 0.08).

### 3.4. Maternal HFD and Antibiotic Treatment Impact the Intestinal Microbiome in Mice

Given the changes in susceptibility of metabolic disease in offspring of HFD-fed mothers, we next began to investigate potential underlying causative traits. Obesity and metabolic disease are linked with altered microbiome composition [50,52,91,92]. Thus, we first examined the effects of maternal and offspring consumption of HFD on offspring fecal microbiome composition (Figure S2A). Prior to offspring dietary challenge, antibiotic treatment was employed to deplete the microbiome. Overall microbial diversity was similar between offspring of CD- and HFD-fed dams at 8 weeks of age (Figure S2B, groups (a) and (b)). However, antibiotic treatment reduced diversity in offspring of HFD-fed dams (Figure S2B, group (d)) in comparison to offspring of CD-fed dams at baseline (group (a)). Reduced diversity was also seen in offspring of HFD-fed dams after 6 weeks of HFD (group (e)) in comparison to group (a). Maternal HFD, antibiotic treatment, and direct HFD feeding all caused qualitative changes in relative abundance of commensal bacteria genera (Figure S2C). Maternal HFD feeding (group (b)) resulted in greater relative abundance of the genera *Muribaculum* and *Bacteroides* and lower *Lactobacillus* compared to maternal CD feeding (group (a), Figure S2C). Antibiotic treatment (group (d)) resulted in higher relative abundance of *Bacteroides* and *Akkermansia* compared to non-antibiotic-treated counterparts (group (c)). HFD-fed offspring of HFD-fed dams (group (e)) had higher relative abundance of *Lactococcus*, *Lactobacillus,* and *Helicobacter* but lower *Bacteroides* compared to the CD-fed offspring of CD-fed dams (group (a)). Antibiotic-treated, HFD-fed offspring (group (f)) had greater relative abundance of *Bacteroides* and lower relative abundance of *Lactobacillus* and *Lactococcus* compared to non-antibiotic-treated, HFD-fed offspring (group (e)). PCA analysis of groups (a) and (b) showed that maternal diet skewed microbiome composition (Figure S2D). Similarly, comparison of groups (c) and (d) showed that after antibiotic treatment, microbiome composition diverged substantially from untreated counterparts (Figure S2E). Following 6 weeks of HFD feeding, the microbiome composition of antibiotic-treated (group (f)) and untreated (group (e)) offspring of HFD-fed dams became similar (Figure S2F). 

**Figure 4:**
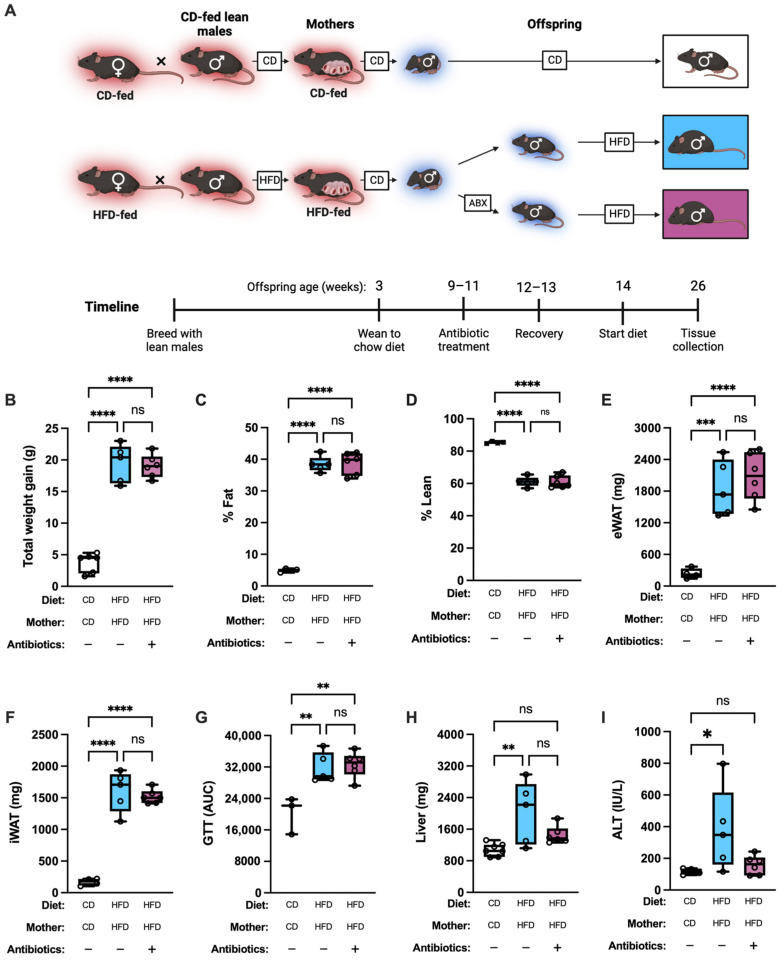
Microbiome manipulation does not influence response to HFD challenge in offspring of HFD-fed mothers. (**A**) Schematic of approach. Red background denotes thermoneutral housing temperature, and blue background denotes thermo-stressed housing. Groups are male offspring of CD-fed or HFD-fed dams. Unless otherwise noted, CD-CD-Abx−, *n* = 4; HFD-HFD-Abx−, *n* = 5; HFD-HFD-Abx+, *n* = 6. (**B**) Weight gain of offspring after 12 weeks on diet. CD-CD-Abx−, *n* = 6. (**C**) Fat percentage of offspring. HFD-HFD-Abx+, *n* = 5. (**D**) Lean mass percentage of offspring. HFD-HFD-Abx+, *n* = 5. (**E**) eWAT mass of offspring. (**F**) iWAT mass of offspring. (**G**) Area under curve (AUC) from two-hour glucose tolerance test (GTT). CD-CD-Abx−, *n* = 3. (**H**) Liver weight of offspring. CD-CD-Abx−, *n* = 7. (**I**) ALT of offspring. (**B**–**I**) One-way ANOVA with Dunnett’s multiple comparisons test. * *p* < 0.05; ** *p* < 0.01, *** *p* < 0.001, **** *p* < 0.0001, ns = not significant.

Whether maternally transmitted adiposity or predilection for metabolic derangement could be modified by the observed changes in the intestinal microbiome was examined next (Figure 4A). 

Antibiotic treatment before HFD feeding was not sufficient to alter total weight gain, adiposity, lean body mass percentage, or glucose tolerance in offspring of HFD-fed dams, compared to untreated offspring of HFD-fed dams (Figure 4B–G). However, unexpectedly, the untreated HFD-fed group had significantly increased liver weight and ALT after 12 weeks of HFD feeding compared to CD-fed offspring of CD-fed dams. In the antibiotic-treated HFD group, no difference was seen in these parameters compared to CD-fed offspring of CD-fed dams (Figure 4H,I).

### 3.5. Maternal Obesity-Associated Inflammation Transfers to the Fetus in Utero in Mice

**Figure 5:**
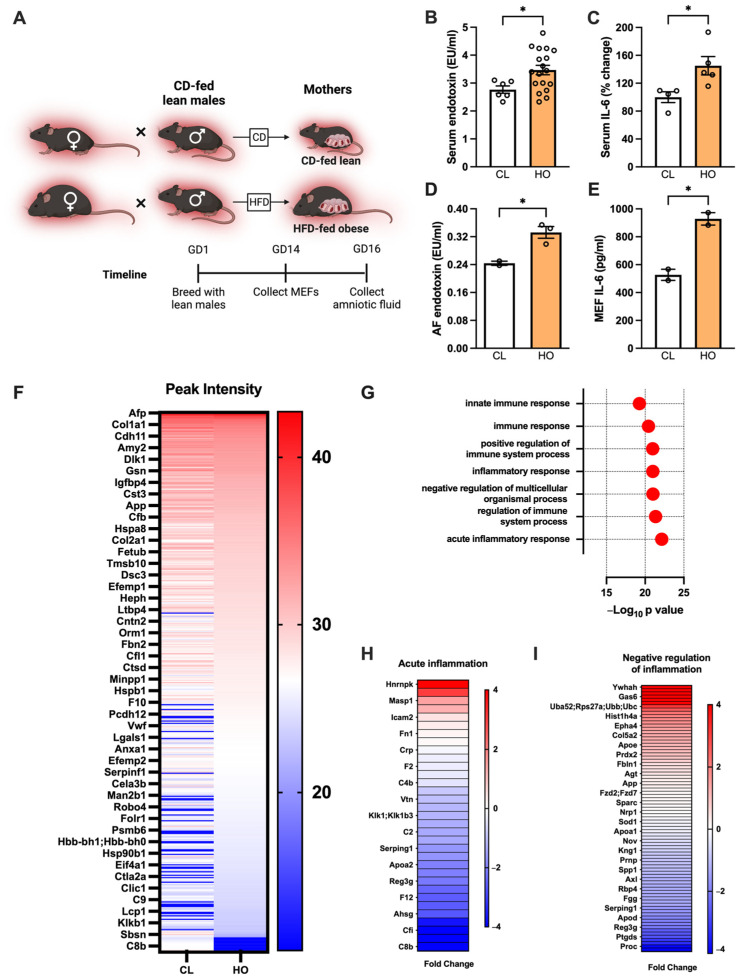
Maternal obesity-associated inflammation transfers to the fetus in utero. (**A**) Schematic of approach. Red background denotes thermoneutral housing temperature. (**B**) Maternal endotoxin at day 16 of pregnancy. Dams are CD-fed lean (CL, *n* = 6) or HFD-fed obese (HO, *n* = 18). (**C**) Maternal serum IL-6 depicted as % change from the average lean female value. CL, *n* = 4; HO, *n* = 5. (**D**) Amniotic fluid endotoxin concentration at GD16. CL, *n* = 2; HO, *n* = 3. (**E**) MEF IL-6 production in response to LPS stimulation. CL, *n* = 2; HO, *n* = 2. (**F**) Proteins differentially present in lean or obese amniotic fluid, represented by peak intensity. (**G**) Most significantly differentially regulated pathways based on protein expression data in obese amniotic fluid compared to lean. (**H**,**I**) Log fold change of proteins in obese amniotic fluid depicted as fold change compared to lean, involved in (**H**) acute inflammation pathway or (**I**) negative regulation of inflammation pathway. (**B**–**E**) Two-tailed *t*-test. * *p* < 0.05.

The intrauterine environment influences offspring health [6,93]. We therefore asked whether the chronic inflammation associated with obesity [94] could be transmitted to the uterine environment and into the fetus. We first investigated whether HO dams exhibited increased inflammation at day 16 of gestation, corresponding to the beginning of the third trimester (Figure 5A).

Serum levels of endotoxin and IL-6 were elevated in HO dams compared to CL dams (Figure 5B,C). Similarly, HO dams displayed increase in amniotic fluid (AF) endotoxin levels compared to CL dams (Figure 5D). We next asked whether the observed increase in gestational inflammation could impact fetal tissue inflammatory responses. Fibroblasts isolated from the embryos of HO dams produced more IL-6 in response to LPS (endotoxin) stimulation compared to those from CL dams (Figure 5E). To begin to understand the obesity-driven differences in gestational environment, preliminary proteomic analysis of AF from an HO dam at day 16 gestation was performed. Notably, AF from an HO dam displayed 128 proteins with differential abundance compared to AF from a CL dam (Figure 5F). Pathway analysis of differentially abundant proteins revealed an emphasis on immune and inflammatory processes (Figure 5G). Proteins related to acute inflammation were overall less abundant in HO compared to CL AF (Figure 5H), while the abundance of proteins linked to negative regulation of inflammation was variable (Figure 5I). 

Whether the inflammation associated with maternal obesity is associated with immunological changes in offspring is not fully understood. To conduct a preliminary study of this question, we compared CD-fed offspring of CL and HO dams (see Figure 2A). Total peripheral white blood cell (WBC) count was similar between the groups at 8 weeks of age (Figure S3A), but offspring of HO dams had significantly altered leukocyte composition in peripheral blood (Figure S3B) and significantly lower frequency of T cells in eWAT (Figure S3C). However, 12 weeks of HFD feeding of offspring from CL or HO dams was sufficient to overcome the differences seen in peripheral leukocyte and eWAT immune cell populations at 8 weeks of age (Figure S3D–F). 

### 3.6. Human Mothers with Obesity Have Altered Inflammation, and Their Infants Experience Differential Weight Gain after Birth

**Figure 6:**
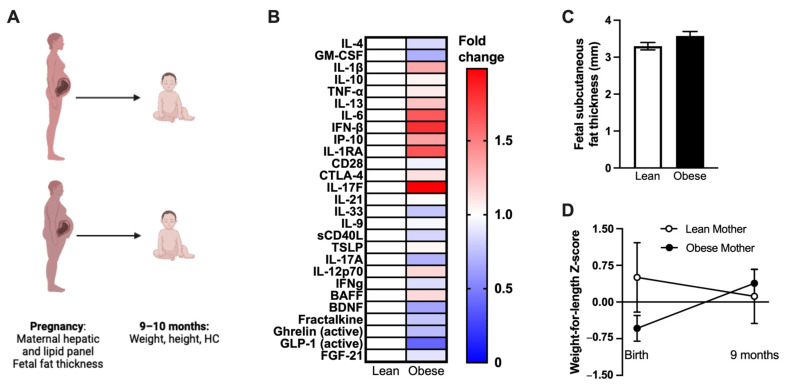
Human mothers with metabolic disease have altered inflammation, and their infants experience differential weight gain after birth. (**A**) Schematic of clinical approach. (**B**) Cytokine levels in maternal serum in third trimester of pregnancy (Lean, *n* = 4; Obese, *n* = 12). (**C**) Fetal subcutaneous fat thickness in third trimester of pregnancy, as measured by MRI (Lean, *n* = 11; Obese, *n* = 30). (**D**) Infant weight-for-length Z-score change from birth to the second study visit at approximately 9 months of age. Lean Mother: r^2^ = 0.01, m = −0.05, non-zero slope *p* = 0.78; *n* = 9. Obese Mother: r^2^ = 0.13, m = 0.12, non-zero slope *p* = 0.03; *n* = 22. (**C**,**D**) Mean ± SEM. (**C**) Two-tailed *t*-test. (**D**) Linear regression.

Lastly, we sought to investigate the clinical utility of our experimental mouse model findings by analyzing the effects of obesity on maternal pregnancy outcomes, maternal inflammation, and early-life infant health in humans. We first analyzed data from a birth cohort study in progress, recruiting mothers with and without obesity. Maternal data were collected during the third trimester of pregnancy and at 9 months postpartum. Infant data were collected at birth and 9 months of age (Figure 6A). 

Mothers were stratified into groups based on pre-pregnancy BMI. As expected by the study design, mothers with obesity had significantly higher pre-pregnancy, pregnancy, and post-pregnancy BMI than mothers without obesity (Table 2). 

Prevalence of pre-pregnancy MASLD or T2D and prevalence of gestational diabetes (GDM) were not significantly different between groups (Table 2). Further, mothers with obesity had significantly lower LDL-C than mothers without obesity in the third trimester of pregnancy. However, no significant differences between groups in other lipid markers (HDL-C, TG, TC) or liver enzymes (bilirubin, albumin, AST, ALT, ALP, total protein) were observed (Table 3). 

We next examined if obesity was associated with maternal inflammation. Cytokines previously associated with increased inflammation, obesity, and metabolic diseases (IL-1β, IL-6, IFN-β, IL-1RA, and IL-17F) were numerically higher but not significantly different in mothers with obesity compared to mothers without obesity (Figure 6B). Finally, the impact of maternal obesity on offspring health outcomes in this cohort was examined. Magnetic resonance imaging (MRI) of fetuses from mothers with obesity showed slightly but not significantly increased fetal subcutaneous fat thickness in the third trimester of pregnancy (Figure 6C). In addition, gestational age at birth was not different between groups of infants from mothers with and without obesity. Further, the proportion of infants with birth weights appropriate for gestational age (AGA) was similar between mothers with and without obesity (Table 4). 

However, from birth to 9 months of age, the average weight-for-length Z-score of infants from mothers with obesity increased by 0.92 ± 0.27 (*p* = 0.03 for test of non-zero change). Multiple regression analysis revealed that the change in weight-for-length Z-score was not due to variation in birth weight, infant sex, or maternal GDM. The study cohort contained only two 9-month measurements for infants of mothers without obesity, but their average weight-for-length Z-score decreased by 0.34 (Figure 6D).

## 4. Discussion

Here, we demonstrate that maternal obesity induced by a combination of HFD feeding and TN housing coupled to syngeneic pregnancy enables studies focused on the discovery of maternal factors contributing to vertical transmission of obesity and obesity-associated metabolic diseases. Using this model in which severely obese mothers weighed 1.5 times more than lean mothers, we show that neonatal offspring of severely obese mothers have significantly decreased survival. Further, preliminary studies with our model of severe maternal obesity suggest that it can be used to study mechanisms of vertical transmission of obesity and metabolic disease. Specifically, we show that offspring of severely obese mothers have increased predisposition to obesity and metabolic dysfunction in adulthood. Notably, we observe that these effects of maternal obesity on offspring metabolic health are independent of offspring microbiome. In addition to metabolic parameters, we also show that obese dams and their fetuses display heightened inflammation during gestation. Lastly, consistent with mouse studies, we show that human mothers with obesity have altered serum cytokines, and their offspring have increased propensity to gain weight within the first year of life. 

Severe obesity (body mass index (BMI) > 35 kg/m^2^) affects 6–10% of adolescents in the United States and is associated with multiple metabolic co-morbidities [95]. Severe obesity continues to rise among youth and was further exacerbated by the COVID-19 pandemic [96]. One factor contributing to this rapid rise in obesity is the transmission of non-genetic predisposing traits from parents to offspring. Although mechanisms of paternal contribution of such traits have been well defined [43], the mechanisms of maternal-specific transmission are more complex and remain insufficiently understood [97,98,99,100,101]. In humans, this is clinically relevant, as the detrimental impact of maternal BMI on offspring health becomes more severe at very low or high BMI values, a phenomenon known as the “U-shaped curve” [102,103,104]. Thus, in this study, we introduce a novel model of severe maternal obesity in WT C57BL/6 mice that allows for the interrogation of maternal pregnancy and offspring health outcomes at the far edge of the U-shaped curve. Coupling our studies with those interrogating pregnancy during maternal starvation [105,106,107] will reveal the mechanistic underpinning of the U-shaped curve. 

Our study recapitulates numerous phenotypic findings from previous work utilizing short- or long-term HFD feeding without severe maternal obesity and at conventional housing temperatures, including increased offspring adiposity and weight gain with consumption of obesogenic diet [108,109,110,111]. Offspring of obese dams developed glucose dysmetabolism after 10 weeks of HFD feeding, while our previous studies demonstrate that C57BL/6J mice born to lean dams typically take 20 weeks on HFD to develop such metabolic perturbations [20]. We also noted a more severe offspring phenotype with use of our severely obese maternal model, including increased offspring mortality during or shortly after birth and in the first few days of life. This phenotype has been scarcely described in mouse models of DIO [112] but closely mimics the human phenotype of increased risk of stillbirth in mothers with obesity—something that adds to the growing list of examples where TN housing allows for a closer recapitulation of human physiology and disease (e.g., cancer [72,73], atherosclerosis [21,74], asthma [75], liver disease [77], and influenza [78]). In fact, increased offspring mortality in severely obese mothers is likely related to insufficient intrauterine nutrition [58,113], while increased mortality within the first few days of life is likely due to impaired lactation or mothering behavior [114,115]. To strengthen the correlation between human outcomes and our mouse model of severe maternal obesity, additional cross-fostering studies are needed to isolate effects of prenatal and postnatal HFD feeding and/or severe maternal obesity on differential fetal/neonatal survival.

Importantly, induction of severe maternal obesity may uncover additional aspects of mechanisms contributing to vertical transmission that remain undetected in existing models of mild obesity. For example, we show increased systemic inflammation in obese female mice housed at TN, aligning with human studies identifying increased serum endotoxin and IL-6 in mothers with obesity [55]. Further, we show significant differences in amniotic fluid of severely obese female mice with increased endotoxin and altered proteome. Such findings add to the highly limited existing analysis of uterine microenvironment to date, with one study reporting increased CRP and TNFα [116], and provide a starting point for mechanistic analyses. For example, future studies utilizing embryo transfer will aid in deciphering contributions of genetic and uterine environment to induction of increased offspring adiposity, as studied previously with outbred mice [117]. The model described here would allow the use of transgenic mice, which are typically generated on C57BL/6 backgrounds, to further explore maternal inflammation as a mechanism of vertical transmission.

We also preliminarily investigated the gut microbiome as a mechanism enabling vertical transmission of metabolic disease. We found that the microbiome differed significantly in young adult offspring of HFD-fed dams, primarily driven by an increase in a relative abundance of the genera *Muribaculum* and *Bacteriodes* and a decrease in *Lactobacillus*, agreeing with results of previous studies finding increased relative abundance of the *Bacteroides* family in offspring of HFD-fed dams [92]. In addition, HFD feeding caused the antibiotic-treated and untreated offspring microbiome to become similar to each other, an unsurprising finding given the known effects of HFD on microbiome composition and long duration of HFD feeding [50,52,91,92]. Finally, we found that manipulation of offspring microbiome through triple antibiotic treatment did not mitigate or worsen the effects of maternal HFD on susceptibility to weight gain and glucose dysmetabolism. However, antibiotic-treated offspring of HFD-fed dams alleviated the increases in liver weight and ALT observed in their untreated counterparts (see Figure 4), which suggests that the microbiome may play a role in HFD-mediated liver pathology. These results are in agreement with the previously described link between the microbiome and MASLD development [8]. Together, our preliminary findings suggest that mechanisms in addition to microbiome modulation contribute to the transmission of obesity and metabolic disease predisposition to offspring. However, formal analyses using germ-free mice (which do not become obese with HFD feeding) colonized with microbes from offspring of severely obese mothers [118] are required for conclusive mechanistic interrogation of the effect of microbiome on vertical transmission of obesity and associated pathology.

Lastly, we analyzed data from a pre-existing human cohort to underscore the mouse model’s relevance to human diseases. Humans typically maintain thermoneutrality by adjusting housing temperature or modifying amount of clothing used, and thus do not need to expend substantial energy to maintain body temperature homeostasis. We show altered systemic cytokine balance in mothers with obesity, adding to existing literature in the field describing increased prenatal TNFα in mothers with obesity compared to mothers without obesity [56]. Additionally, our finding of increasing weight-for-length Z-score in infants born to mothers with obesity may provide insight into offspring predisposition to metabolic disease. Weight gain causing an increase of more than 0.67 Z-score units in the first two years of life has been linked to increased adiposity later in childhood [119]. Strikingly, our studies indicate an average change in weight-for-length Z-score of +0.92 units from birth to 9 months of age in infants of mothers with obesity. Future studies will seek potential connections between intrauterine inflammation, offspring rapid growth and adiposity, and predisposition to diet-induced obesity and metabolic disease.

Given our study premise to propose a novel experimental model that can be used to study vertical transmission of obesity and obesity-associated metabolic/inflammatory dysfunction, we included relatively small sample sizes of mice that may have limited power to fully replicate previously published findings. The small sample sizes were also due to poor survival of offspring of HO dams. Similarly, only male offspring were used for studies due to the need to propagate the colony using female offspring. Future studies should explore vertical transmission of obesity and metabolic disease predisposition in female offspring alongside male offspring. Notably, we found elevated adiposity but no glucose dysmetabolism or liver damage in CD-fed, 8-week-old offspring of obese dams. As some studies in mice and rats note increased weight and metabolic disease in middle-aged or older offspring of HFD-fed mothers, even when they remain on CD [110,120,121], further studies are needed to determine whether middle-aged offspring in our model also develop metabolic dysfunction. In addition, the association between maternal obesity and dysregulated offspring immunity has been strongly supported in rodents, non-human primates, and humans [54], with both maternal obesity and obesogenic diet shown to decrease WBC count [122,123]. We noted only a trend toward decreased WBC count in offspring of HO dams compared to that of CL dams, but the differences were eliminated after 12 weeks of HFD feeding. One possible explanation for this result is that prolonged HFD feeding may mask any lasting effect from maternal obesity on WBC counts. Further studies should utilize diets with a lower fat percentage or higher carbohydrate percentage, as well as earlier endpoints, to uncover early drivers of disease in offspring of obese dams. Given the challenges in securing the numbers of obese dams and offspring, our studies were also restricted with the experimental numbers used for analyses of amniotic fluid endotoxin measurements and MEF IL-6 production. Finally, the small size of our human cohort also limited the statistical power of our analyses. While large studies have described an increased risk for infants from mothers with obesity to be small or large for their gestational age (SGA/LGA; [113]), we found equal percentages of average-for-gestational-age infants in mothers with and without obesity in our cohort.

Our study also highlights avenues of further investigation of several potential mechanisms of vertical transmission of metabolic disease. Epigenetic modification is linked with offspring obesity predisposition [6]; yet, despite such relevance, our studies did not investigate whether maternal obesity promotes epigenetic modifications occurring before conception or during gestation. Similarly, maternal diet during lactation has been shown to impact offspring weight and metabolic function [124]. However, in our studies, we did not separate the effects of maternal HFD or obesity during lactation versus gestation. Conducting embryo transfers and/or cross-fostering studies in association with our model would address these two points. Lastly, our studies were done on inbred mice with an identical genetic background. Importantly, differences in ethnic background are present in humans and linked to differences in maternal morbidity and mortality, as well as differential metabolic disease risk between ethnic groups [125]. Hence, we recognize that allogeneic mating studies using HFD feeding combined with TN housing are needed.

## 5. Conclusions

Taken together, our data indicate that the use of thermoneutral housing coupled with HFD feeding and successful pregnancy recapitulates and amplifies key parameters seen in other models of female DIO and vertical transmission of obesity and metabolic disease. Further, this model of maternal obesity promotes the activation of inflammatory processes in gestational tissues. As such proinflammatory skewing may represent an important locus of vertical transmission of metabolic diseases, the inflammatory milieu associated with severe maternal obesity should be investigated as a mechanism of transmission and could represent an avenue for future interventions. Thus, the key strength of this model is its ability to induce a more severe maternal phenotype of both obesity and metabolic disease, more closely mimicking “human-like” responses. As such, this model may be used to uncover critical mediators not previously appreciated in other experimental models.

## Figures and Tables

**Table 1:** Effects of various obesogenic diets on parameters associated with obesity and metabolic disease in C57BL/6 female mice.

Parameter	HFD (Ts)	HF-HC/HF-HS	Cafeteria	HFD or WD (Tn)
Weight gain	30–50% increase [20,22,23]	50% increase [27,28,29]	30–60 + % increase [30,31,32]	100 + % increase [20]
GTT	~50% increase [22,23]	50–100% increase [27,28]	Studies needed, glucose high [31]	100% increase [20]
Liver weight	No change [20]	Increased [33]	Increased [31]	Increased [20]
Liver triglyceride	No change [20]	Studies needed	Increased [31]	Increased [20]
ALT	No change [20]	Increased [33]	Increased [31]	Increased [20]
Serum triglyceride	Increased [22]/ not increased [25]	Increased or decreased [33,34]	Increased [30,32]	Studies needed *
Serum LDL	Mildly increased [26]	No change or increased [29,33]	No change [23,25]	Studies needed *
Atherosclerotic lesions	None [26]	None [29]	Studies needed	Studies needed *

* Studies were conducted in male mice by our group [21] but not in females.

**Table 2:** Maternal cohort characteristics. Data shown as mean ± SD or n (%), with asterisk denoting significant difference from lean group (* *p* < 0.05; Fischer’s exact test or *t*-test, as appropriate).

Parameter	Lean (*n* = 11)	Obese (*n* = 30)
Age (years)	30 ± 4	30 ± 6
White race	11 (100%)	22 (73%)
Family history of NAFLD	1 (9%)	3 (10%)
Pre-pregnancy NAFLD	0 (0%)	5 (17%)
Pre-pregnancy T2D	0 (0%)	5 (17%)
Gestational diabetes	2 (18%)	9 (30%)
Bariatric surgery history	0 (0%)	2 (7%)
Insulin use in pregnancy	0 (0%)	7 (23%)
Multivitamin use in pregnancy	8 (73%)	7 (23%) *
Pre-pregnancy BMI (kg/m^2^)	22.2 ± 2.9	39.2 ± 8.8 *
Pregnancy BMI (kg/m^2^)	25.2 ± 2.7	41.2 ± 7.9 *
Pregnancy number	3 ± 2	3 ± 3
Living children	1 ± 1	1 ± 1
C-section delivery	5 (45%)	12 (40%)
BMI at 9 months (kg/m^2^)	20.2 ± 2.4	38.1 ± 8.8 *

**Table 3:** Maternal lipid profile and hepatic function panel. Data shown as mean ± SD, with asterisk denoting significant difference from lean group (* *p* < 0.05; *t*-test).

Parameter	Lean (*n* = 10)	Obese (*n* = 28)
HDL (mg/dL)	67 ± 15	61 ± 12
TG (mg/dL)	199 ± 39	210 ± 71
LDL (mg/dL)	153 ± 46	122 ± 34 *
TC (mg/dL)	237 ± 49	225 ± 44
Total bilirubin (mg/dL)	0.34 ± 0.21	0.32 ± 0.12
Albumin (g/dL)	2.9 ± 0.2	2.8 ± 0.2
AST (units/L)	21 ± 4	24 ± 29
ALT (units/L)	13 ± 7	28 ± 56
ALP (units/L)	108 ± 35	125 ± 36
Protein (g/dL)	6.3 ± 0.3	6.4 ± 0.4
One-hour GCT (mg/dL) ^1^	109 ± 16	122 ± 26

^1^ For GCT: Lean, *n* = 8; Obese, *n* = 21.

**Table 4:** Infant characteristics. Data shown as mean ± SD or n (%). No significant difference from lean group (Fischer’s exact test or *t*-test, as appropriate).

Parameter	Lean (*n* = 10)	Obese (*n* = 29)
White race ^1^	8 (89%)	21 (75%)
Male sex	5 (50%)	13 (45%)
Gestational age at birth (weeks)	39 ± 1	38 ± 1
Birth weight within AGA range ^2^	9 (82%)	25 (83%)

^1^ For white race: Lean, *n* = 9; Obese, *n* = 28. ^2^ For birth weight within AGA range: Lean, *n* = 11; Obese, *n* = 30.

## Data Availability

The data presented in this study are available on request from the corresponding author. Data are not publicly available due to privacy.

## Supplementary Materials

The following supporting information can be downloaded at: https://www.mdpi.com/article/10.3390/nu15234958/s1, Figure S1: Maternal obesity does not affect pregnancy outcomes; Figure S2: Maternal diet, antibiotic treatment, and HFD feeding impact offspring microbiome; Figure S3: Maternal obesity impacts offspring immunological characteristics.
