# Supplementary material for: Thermoneutral Housing Enables Studies of Vertical Transmission of Obesogenic Diet-Driven Metabolic Diseases
Source: Nutrients. 2023 Nov 29;15(23):4958. doi: 10.3390/nu15234958 (PMC10708424; doi:10.3390/nu15234958)
Supplement: Supplementary file 1 [file nutrients-15-04958-s001.zip › nutrients-2714160-supplementary.pdf]

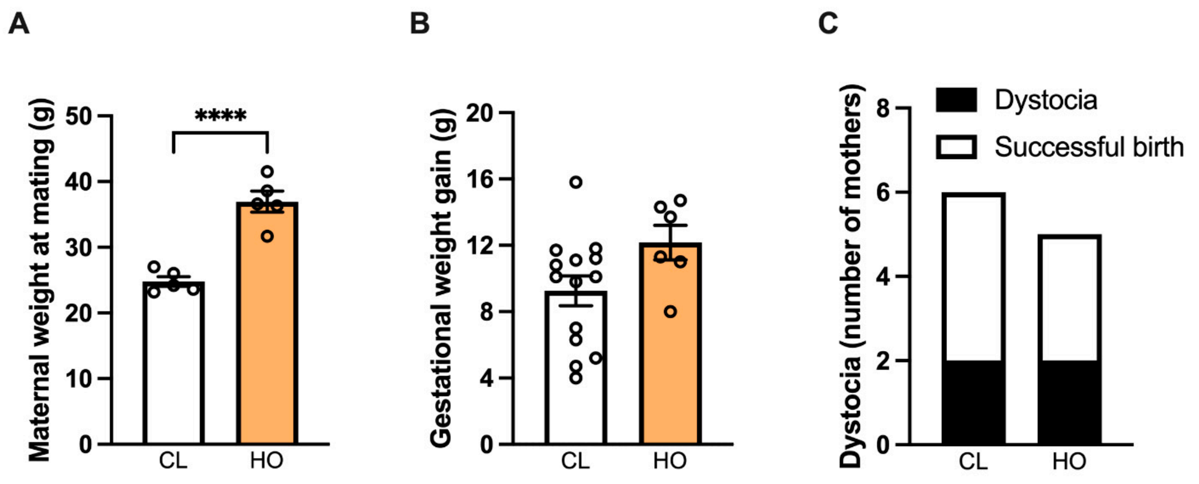

Supplementary Figure S1: Maternal obesity does not affect pregnancy outcomes.

(A) Maternal weight at mating in the CD-fed lean (CL, n = 5) and HFD-fed obese (HO, n = 5) groups. (B) Gestational weight gain (CL, n = 14; HO, n = 6). (C) Number of first-time dams experiencing dystocia (CL, n = 6; HO, n = 5). (A, B) Unpaired t-test. **** $p < 0.0001$. (C) Chi-square test for trend, $p = 0.85$.

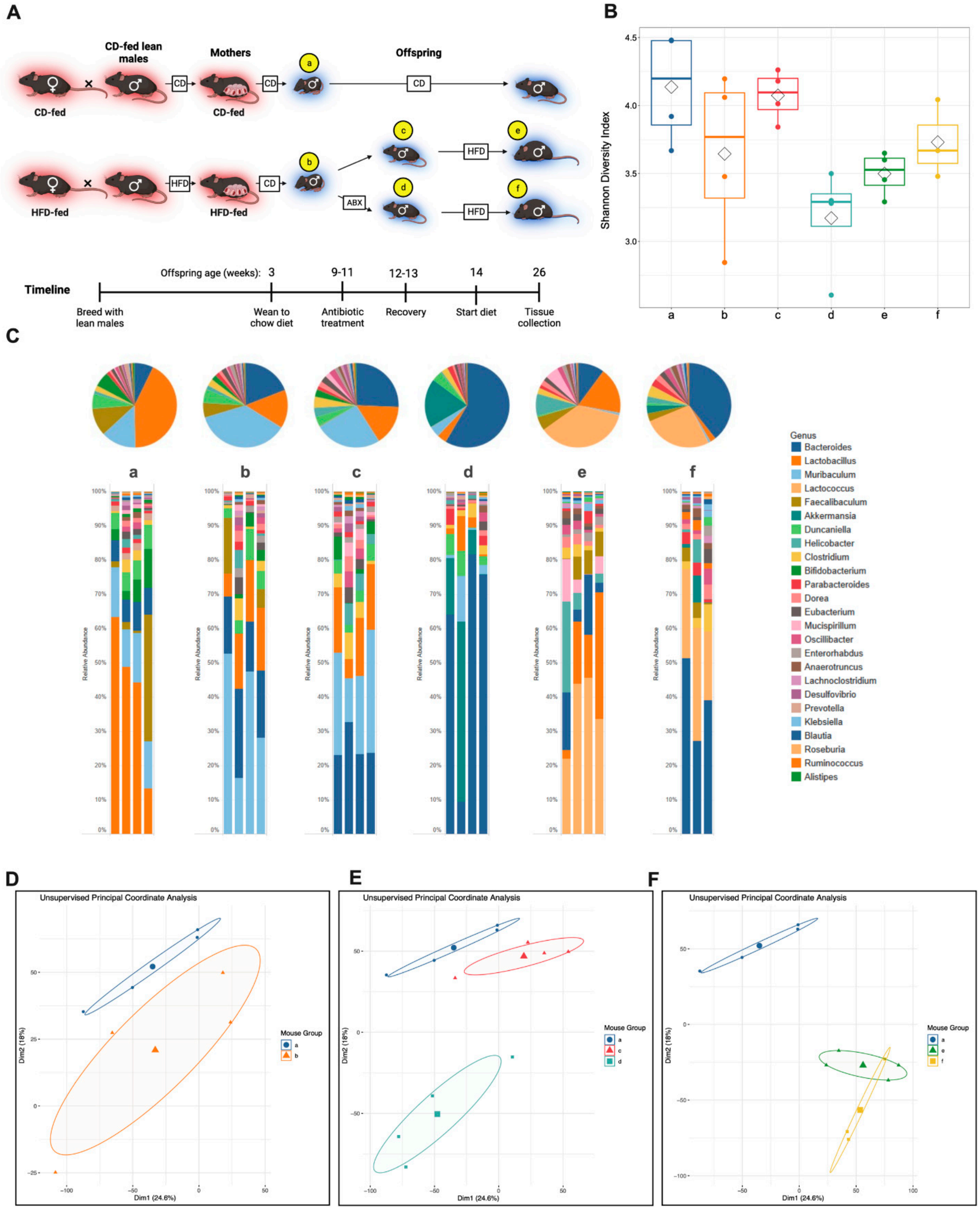

A
CD-fed lean males
Mothers
Offspring
CD-fed
CD-fed
HFD-fed
HFD-fed
CD
HFD
ABX
a
b
c
d
e
f
Timeline
Offspring age (weeks): 3
9-11
12-13
14
26
Breed with lean males
Wean to chow diet
Antibiotic treatment
Recovery
Start diet
Tissue collection
B
Shannon Diversity Index
C
Relative Abundance
Genus
Bacteroides
Lactobacillus
Muribaculum
Lactococcus
Faecalibaculum
Akkermansia
Duncaniella
Helicobacter
Clostridium
Bifidobacterium
Parabacteroides
Dorea
Eubacterium
Mucispirillum
Oscillibacter
Enterorhabdus
Anaerotruncus
Lachnoclostridium
Desulfovibrio
Prevotella
Klebsiella
Blautia
Roseburia
Ruminococcus
Alistipes
D
E
F
Unsupervised Principal Coordinate Analysis
Dim1 (24.6%)
Dim2 (18%)
Mouse Group

Supplementary Figure S2: Maternal diet, antibiotic treatment, and HFD feeding impact offspring microbiome.

(A) Schematic of approach. Fecal samples were collected from the labeled groups and sequenced for intestinal microbiome analysis: [a] 8-week-old offspring of CD-fed mothers; [b] 8-week-old offspring of HFD-fed mothers; [c] 13-week-old offspring of HFD-fed mothers; [d] 13-week-old offspring of HFD-fed mothers given antibiotics; [e] offspring of HFD-fed mothers after 10 weeks of HFD; [f] offspring of HFD-fed mothers after antibiotic treatment, then 10 weeks of HFD. n = 4 per group. (B) Shannon Diversity Index of the labeled groups. (C) Representation of intestinal microbiome composition at the genus level. Pie chart represents average among samples; bars represent individual animals. (D) PCA plot representation of intestinal microbiome diversity between groups [a] and [b]. (E) PCA plot representation of intestinal microbiome diversity between groups [a], [c], and [d]. (F) PCA plot representation of intestinal microbiome diversity between groups [a], [e], and [f].

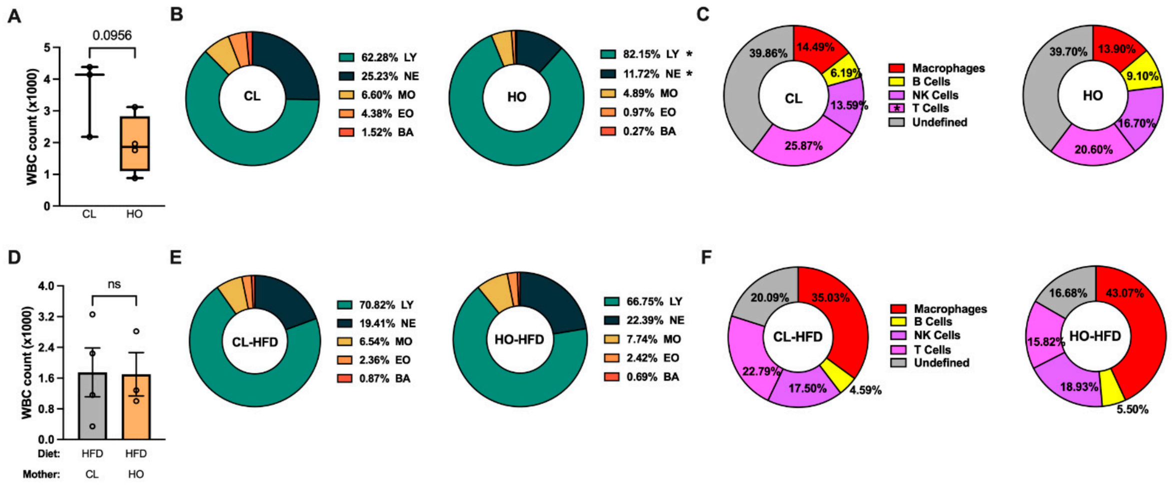

Supplementary Figure S3: Maternal obesity impacts offspring immunological characteristics.

(A) Total peripheral leukocyte count and (B) white blood cell differential, determined by Hemavet, and (C) eWAT immune cell frequencies, determined by flow cytometry, in offspring of CD-fed lean (CL) and HFD-fed obese (HO) mothers. (D) Total peripheral leukocyte count, (E) white blood cell differential and (F) eWAT immune cell frequencies in offspring of CD-fed lean and HFD-fed obese mothers after 12 weeks of HFD. (A, D) Unpaired t-test. (B, E) Chi-square test. * $p < 0.05$, ns = not significant. (C,F) Two-way ANOVA with Sidak's multiple comparisons test. (A-C) CL, $n = 3$; HO, $n = 4$. (D-E) HFD-CL, $n = 4$; HFD-HO, $n = 3$.
